# A Two-Dimensional Layered Bismuth Coordination Polymer Based on a Lone Pair−π Interaction

**DOI:** 10.1021/acs.inorgchem.6c01816

**Published:** 2026-06-12

**Authors:** Haruto Yamaoka, Kazuya Otsubo

**Affiliations:** † Department of Chemistry, Faculty of Science, 183812 Tokyo University of Science, Shinjuku-ku, Tokyo 162-8601, Japan; ‡ Research Institute for Science and Technology, Division of Joint Research of Geometry and Natural Science, Tokyo University of Science, Chiba 278-8510, Japan

## Abstract

Metal–organic frameworks (MOFs) and coordination polymers (CPs), which are made from a wide variety of metal ions and organic ligands, constitute a broad and rapidly developing research field spanning both fundamental studies and applications. Here, we report the synthesis, crystal structure, and electronic properties of Bi­(PCA)­(H_2_O) (H_3_PCA = protocatechuic acid), the first example of a CP based on a cation-based lone pair−π interaction. This CP features a two-dimensional (2D) layered structure composed of Bi^3+^ ions and PCA^3–^ ligands. The central Bi^3+^ ion adopts an unusual hemispherical six-coordinated geometry, attributed to the presence of a stereochemically active 6s^2^ lone pair. Single-crystal X-ray diffraction (XRD) analysis revealed that the 2D layers are assembled in an alternating interdigitated manner through cation-based lone pair−π interaction between the 6s^2^ lone pairs of Bi^3+^ ions and the π-cloud of PCA^3–^ ligands, as well as π–π interaction between adjacent PCA^3–^ ligands. Furthermore, optical absorption measurements and density functional theory (DFT) calculations demonstrated the presence of a characteristic absorption band associated with the lone pair−π interactions between the 2D layers. These findings highlight the potential of exploiting unique coordination geometries of metal ions to design novel MOFs and CPs with distinctive crystal structures and electronic properties.

## Graphical abstract

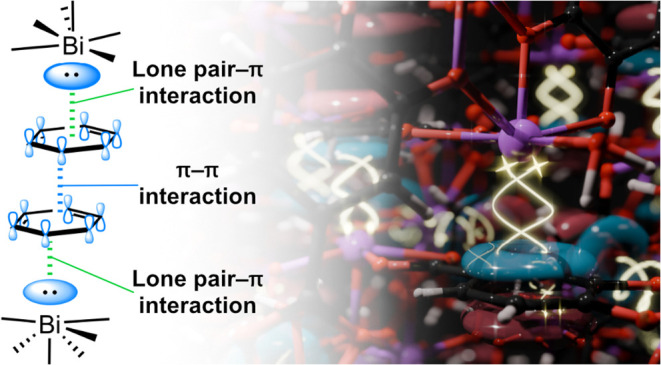

## Introduction

Metal–organic frameworks (MOFs) and coordination polymers (CPs), which are formed through the self-assembly of metal ions and organic linkers with diverse combinations, have emerged as one of the most intensively studied classes of materials in recent years due to their potential applications in catalysis,^1,2^ gas storage,^3,4^ separation,^5^ guest recognition,^6,7^ biomedicine,^8^ proton/electron conduction,^9–13^ and more.^14–17^ A major advantage of these materials lies in the tunable design of their frameworks, which can be achieved by selecting appropriate metal ions and ligands. In particular, MOFs and CPs constructed from early transition-metal ions such as 3d and 4d metals have already established a significant area of research.^18–21^

On the other hand, post-transition metals—especially p-block cations such as Bi^3+^, Pb^2+^, and Sn^2+^—exhibit distinctive coordination geometries that markedly differ from those of lanthanides, where f-orbitals participate in coordination bonds.^22,23^ These p-block elements possess a stereochemically active ns^2^ lone pair, often resulting in hemidirected (hemisphere-like) or holodirected (spherically symmetric) coordination environments.^24^ In the field of condensed matter physics, such elements are also recognized as valence-skipping species.^25^ Among them, bismuth-based MOFs and CPs have attracted particular attention due to bismuth’s low toxicity^26^ and cost-effectiveness, making it a promising candidate for use in active pharmaceutical ingredients (APIs)^27–29^ and low-cost catalysts.^30,31^ Furthermore, Bi-based MOFs and CPs have been actively explored for their applications in luminescent materials,^32–34^ proton conductivity,^35^ and chemical sensing.^36^

From a structural point of view, MOFs and CPs are primarily composed of coordination-bonded network structures; however, in many crystal structures, intermolecular interactions such as hydrogen bonding and π–π interactions also contribute significantly to structural stabilization. Among these, interactions between nonbonding electron pairs (lone pairs) and π-electron clouds—referred to as lone pair−π interactions—are also recognized as a key intermolecular interaction. Nevertheless, examples of lone pair−π interactions in MOFs and CPs remain scarce and are mostly limited to interactions involving anions (primarily halides) or lone pairs on oxygen or nitrogen atoms within ligands interacting with π-systems.^37–40^ Many examples of low-dimensional Bi and Sb compounds, including zero-dimensional (0D) and one-dimensional (1D) systems, have been reported in which cationic lone pair−π interactions are considered to contribute to structural stabilization within the crystal lattice. Representative examples, particularly among Bi-based compounds, include organic–inorganic hybrid materials composed of bismuth halides, as well as mononuclear and dinuclear complexes and cluster compounds.^41^ In contrast, no examples have thus far been reported in which cationic lone pair−π interactions are regarded as stabilizing factors in two-dimensional (2D) layered structures.

Here, we report the synthesis, crystal structure, and electronic properties of a novel two-dimensional (2D) bismuth (Bi)-based CP, Bi­(PCA)­(H_2_O) (H_3_PCA: protocatechuic acid, **1**), stabilized by 6s^2^ lone pair−π interactions. This compound features a 2D layered structure formed from Bi^3+^ ions and PCA^3–^ ligands, with the layers stacked through both π–π interactions and 6s^2^ lone pair−π interactions. This is the first example of a 2D CP in which the cationic ns^2^ lone pair−π interaction contributes to interlayer stacking. We successfully obtained single crystals of **1** suitable for single-crystal X-ray diffraction analysis, allowing unambiguous determination of its characteristic structure. Furthermore, optical spectroscopic observations combined with density functional theory (DFT) calculations confirm the presence of 6s^2^ lone pair−π interactions between the Bi orbitals and the π-electron system of the PCA^3–^ ligands, which play an important role in the stabilization of the layer-stacking-type crystal structure of **1**.

## Results and Discussion

### Synthesis and Characterization

The synthetic scheme of compound **1** is shown in Figure 1. Yellow plate-like single crystals of **1**, with approximate edge lengths of 0.1 mm, were successfully obtained by heating a mixture of Bi­(NO_3_)_3_·5H_2_O and the catechol-based organic ligand, protocatechuic acid (3,4-dihydroxybenzoic acid, H_3_PCA) in a mixed solvent of water and acetic acid at 80 °C (Figure S1). Elemental analysis revealed that Bi^3+^ ions, PCA^3–^ ligands, and water molecules are present in a 1:1:1 ratio. The crystal structure of compound **1** was determined by single-crystal X-ray diffraction (XRD). Figure 2 shows the X-ray crystal structure of **1** at 90 K. **1** crystallizes in the *P*2_1_/*n* space group (no. 14), and the central structural motif comprises a binuclear unit formed by two Bi^3+^ ions, four PCA^3–^ ligands, and two coordinated water molecules, which can be described as a Bi_2_O_10_ core (Figure 2a,b). Within this core, the Bi–O bond lengths range from 2.141(4) to 2.603(4) Å. Considering that typical Bi–O bond lengths range from 2.1 to 3.0 Å, **1** can be regarded as one of the Bi-based MOFs and CPs featuring relatively short Bi–O bond distances, even in comparison with the previously reported compound exhibiting the shortest Bi–O distance (2.137(2) Å).^42^ These Bi_2_O_10_ binuclear units are bridged by PCA^3–^ ligands to form a 2D layered structure (Figure 2c,d). The structural characteristics of the Bi_2_O_10_ core, which serves as the central metal unit of **1**, were also confirmed by spectroscopic measurements. In the Raman spectrum at room temperature (rt), a very intense peak was observed at 108 cm^–1^ (Figure S2). Based on the DFT calculation using a model structure having a single Bi_2_O_10_ core (model 1, see SI), this intense peak was attributed to a symmetric stretching vibrational mode where the Bi_2_O_10_ core undergoes a symmetric distortion. Fourier-transform infrared (FTIR) spectra of a polycrystalline powder sample of **1** (Figures S3 and S4) exhibited a shift of the ν­(C=O) mode of the carboxyl group toward lower wavenumbers relative to that of free H_3_PCA, indicating coordination of the ligand to the Bi^3+^ centers.

**Figure 1:**
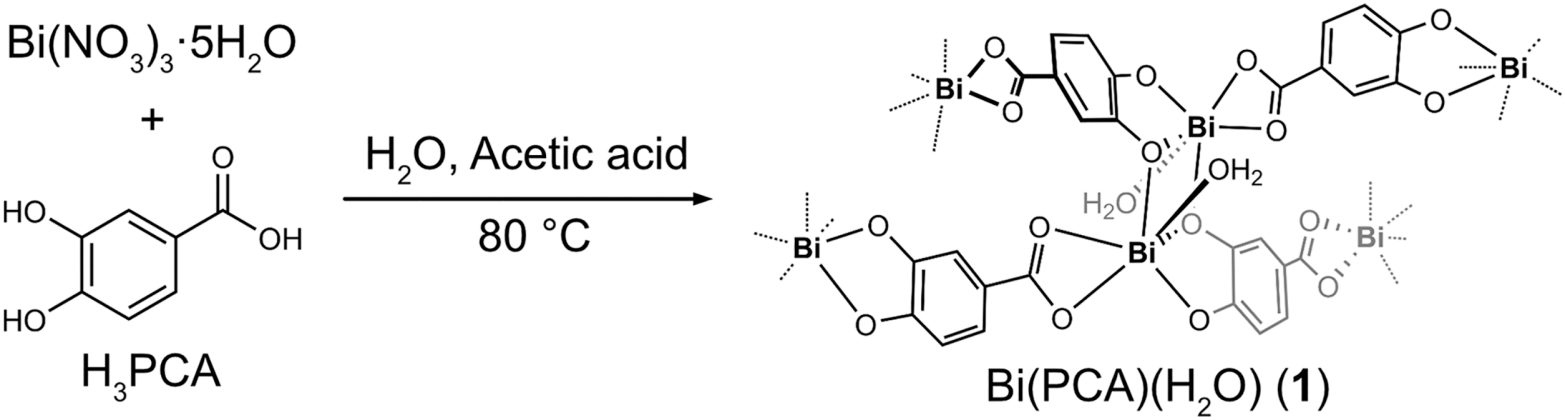
Schematic representation of the synthetic route of **1**.

**Figure 2:**
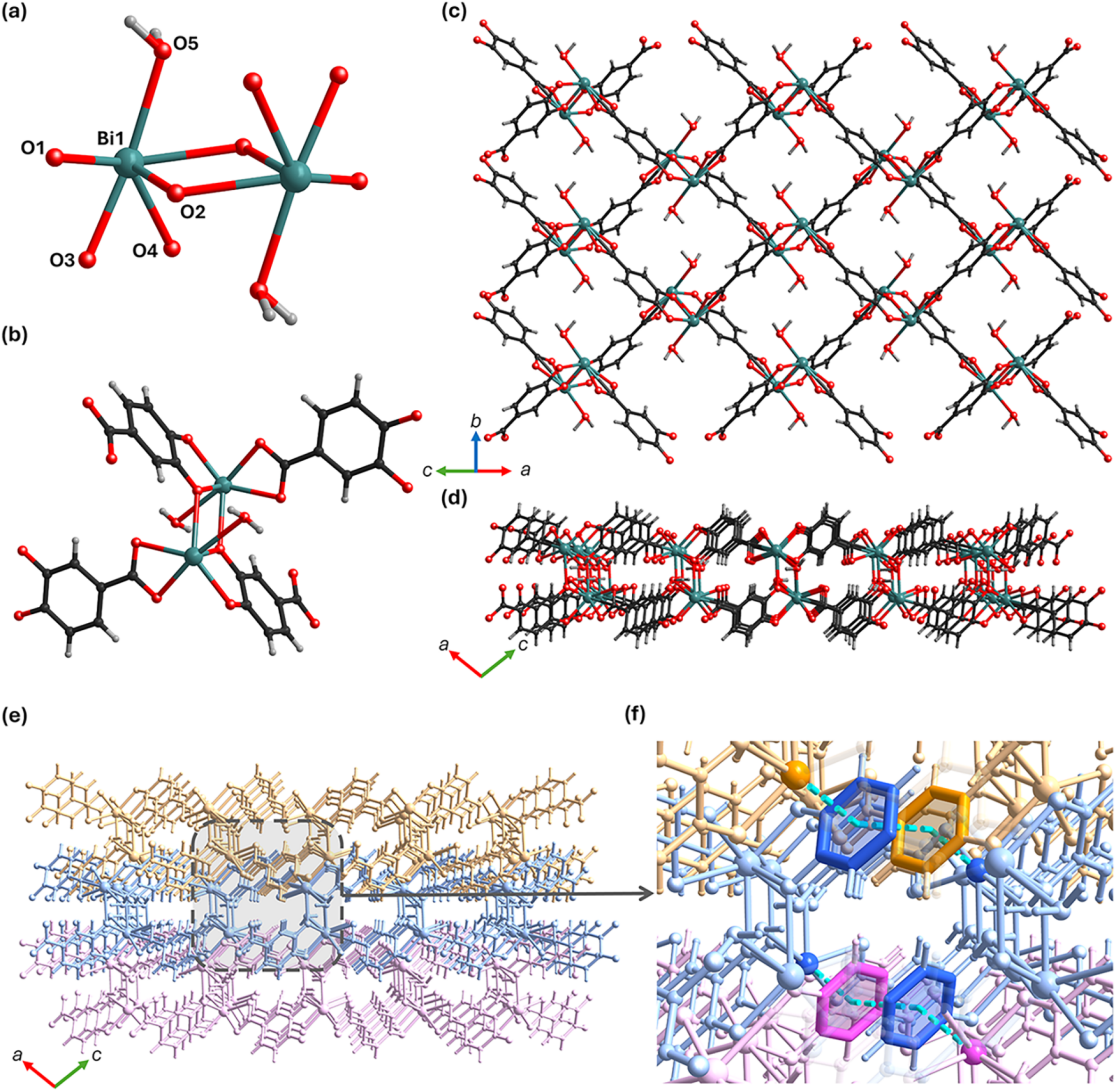
X-ray crystal structure of **1** at 90 K. (a) Coordination geometry around the Bi^3+^ ion. (b) Structure of Bi_2_O_10_ core. (c) Top view of the 2D layered structure. (d) Side view of the 2D layered structure. (e) 3D packing structure based on the layer stacking. An independent 2D layered structure is highlighted in a single color (light yellow, blue, and pink). (f) Schematic representation (depicted from the gray shaded area in panel e) of the interlayer interactions (see text). All panels are drawn in a ball-and-stick model. Bi, C, O, and H atoms are shown in green, gray, red, and white, respectively.

### Lone Pair−π Interaction

Notably, this 2D framework is further assembled into the overall three-dimensional architecture through unique intermolecular interactions, as described below. Focusing on the coordination environment of a single Bi^3+^ ion, it adopts a hemidirected, stereochemically active six-coordinated geometry, with the 6s^2^ lone pair prominently protruding outside.^23,24^ The presence of the stereochemically active lone pair was further confirmed by DFT calculation on model 1 composed of one Bi_2_O_10_ core (HOMO–10, Figure 3a and Table S1). This protruding lone pair lies 3.02 Å away from the aromatic ring of a PCA^3–^ ligand on an adjacent 2D layer along the ***a*** + ***c*** direction (Figure 2d–f). This distance is significantly shorter than the sum of the van der Waals radii of Bi^3+^ and carbon atoms (∼3.7 Å), suggesting that the interlayer cohesion is mediated by lone pair−π interactions. Additionally, strong π–π stacking interactions are observed between opposing aromatic rings of PCA^3–^ linkers on adjacent layers, with an interplanar spacing of 3.10 Å (Figure 3b). This revealed that **1** adopts an interdigitated structure in which the 2D layers are linked through a highly ordered and distinctive stacking arrangement featuring a linear sequence of lone pair−π, π–π, and lone pair−π interactions. To the best of our knowledge, **1** represents the first example of a coordination polymer, where the cation-based lone pair−π interaction plays an important role in the structural stabilization. In general, 2D layered compounds composed of metal ions possessing an ns^2^ lone pair, such as Bi^3+^, are typically constructed from highly coordinated metal centers and ditopic organic ligands, in which the lone pairs on the mononuclear metal centers are often stereochemically inactive.^43,44^ In contrast, in the present compound **1**, the central metal unit forms a dinuclear Bi_2_O_10_ core, in which the stereochemically active lone pairs protrude outward from the core and are thus capable of interacting with adjacent molecules. This unique structural feature is considered to be responsible for the emergence of lone pair−π interactions within the 2D layered structure. In addition, calculations of the void volume using the PLATON software^45^ revealed that **1** contains no significant voids and adopts a dense packing structure. These intermolecular interactions described above are considered to give rise to the dense packing structure of **1**.

**Figure 3:**
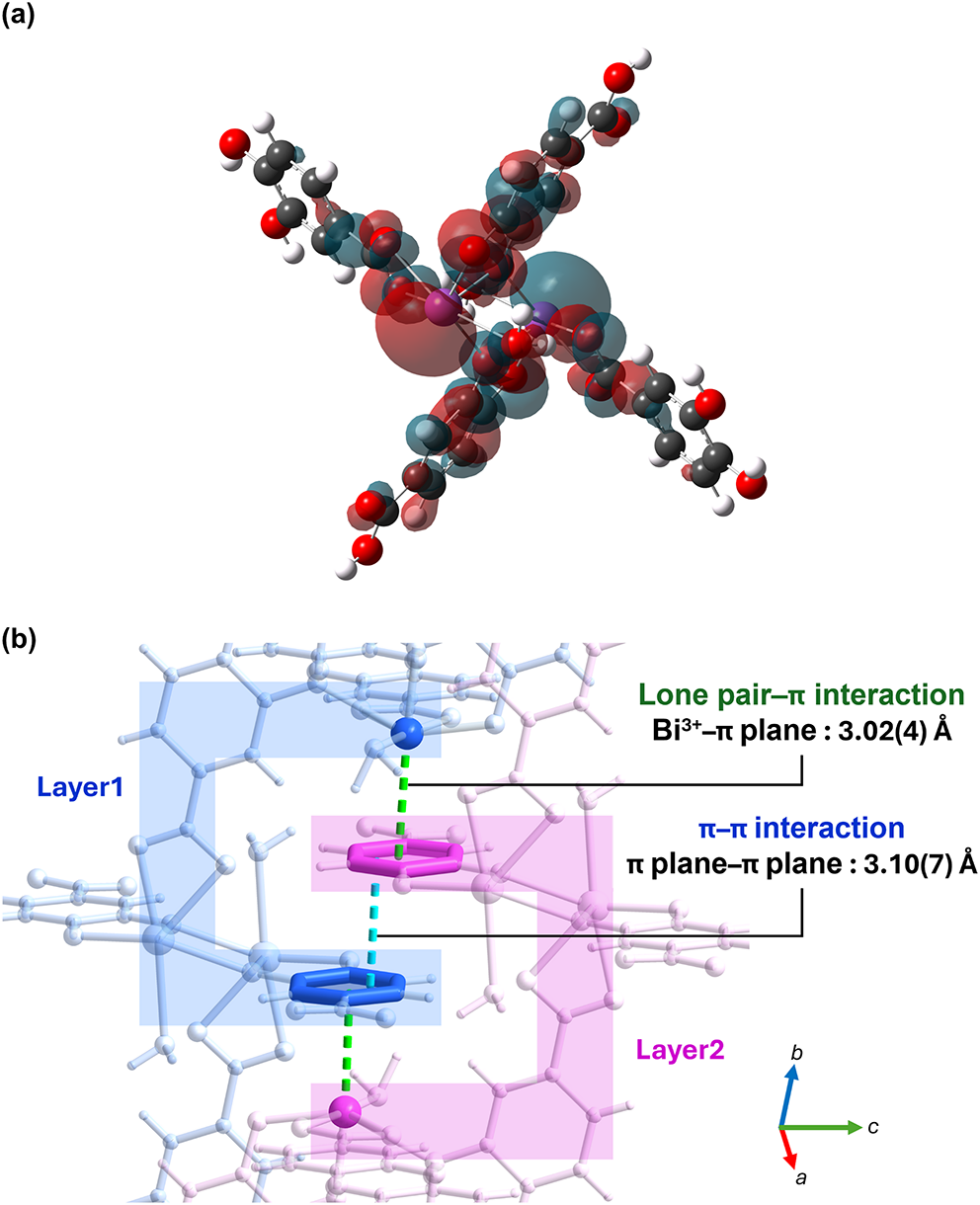
Lone pair−π interaction in **1**. (a) HOMO–10 molecular orbital for model 1 based on the DFT calculation. A spherical s-type lobe is seen at the Bi^3+^ center. (b) Enhanced view of the interlayer interaction. Specific distances estimated from X-ray crystal structure analysis are also shown.

The thermal stability of compound **1** was investigated by thermogravimetric analysis (TGA). As shown in Figure 4, a significant weight loss attributable to decomposition was observed at approximately 290 °C, with no distinct weight-loss step corresponding to the release of coordinated/noncoordinated solvent molecules. This suggests that the coordinated H_2_O molecule is a part of the Bi_2_O_10_ binuclear unit, and its removal would compromise the structural integrity of the unit. On the other hand, as mentioned above, the 2D layered structure in **1** is stabilized by lone pair−π and π–π interactions between adjacent layers, which likely contribute to the overall structural stability up to relatively high temperatures.

**Figure 4:**
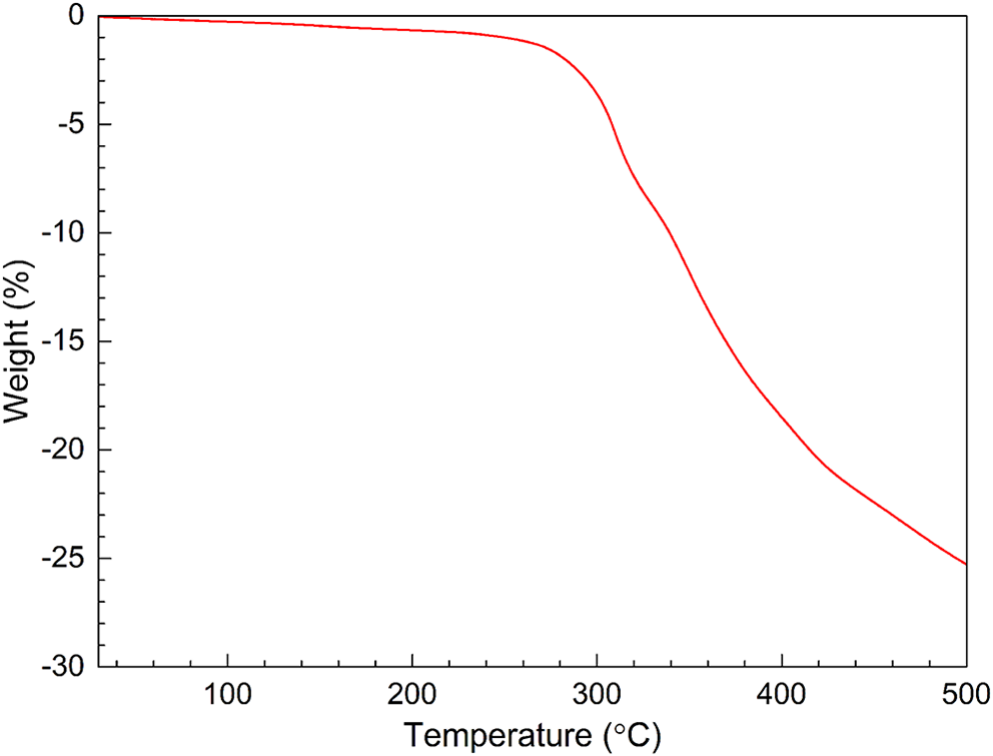
Thermogravimetric analysis of **1**.

On the basis of the TGA results, the thermal stability of **1** was further investigated under heating conditions immediately prior to the onset of the major weight loss (250 °C). However, powder X-ray diffraction (PXRD) measurements revealed that **1** transformed into a different crystalline phase (Figure S5), indicating that the thermal stability of this compound is not sufficiently high to allow the removal of the coordinated H_2_O molecules while retaining the original structure. We further investigated the pH stability of **1** as well as its stability toward various solvents. Polycrystalline powder samples of **1** were immersed for 24 h in aqueous solutions adjusted to various pH values using HCl and NaOH, followed by PXRD measurements (Figure S6). The results revealed that **1** retains its original structure over a wide pH range from 2 to 12. In addition, immersion of **1** in various solvents showed that the structure remains stable in most solvents examined (Figure S7). The remarkable structural stability of **1** over a wide pH range may originate from the relatively robust intermolecular interactions present within the crystal structure, including lone pair−π and π–π interactions, which are considered to stabilize the framework.

### Electronic Property and TD-DFT Calculation

To gain further insight into the lone pair−π interactions in **1**, a time-dependent DFT (TD-DFT) calculation was performed using a model structure that reproduces the interdigitated stacking motif (model 2, Table S3). As an example, Figure 5a shows the HOMO–30 molecular orbital, in which clear overlap can be observed between the prominent lobe of the Bi^3+^ 6s^2^ lone pair and the π-electron cloud of the aromatic ring from an adjacent PCA^3–^ ligand (see also Figure S8). Further analysis of the orbital composition showed that the Bi^3+^-derived contributions to HOMO–30 (Figure 5a) are 0.62% for Bi­(A) and 0.46% for Bi­(B), with s- and p-orbital contributions of 50 and 49% for Bi­(A) and of 43 and 52% for Bi­(B), respectively, indicating sp hybridization. Similarly, orbital overlap was observed between adjacent PCA^3–^ aromatic rings, indicating strong π–π interactions (Figure 5b). These DFT results support the idea that both lone pair−π and π–π interactions contribute significantly to the structural stabilization in **1**.

**Figure 5:**
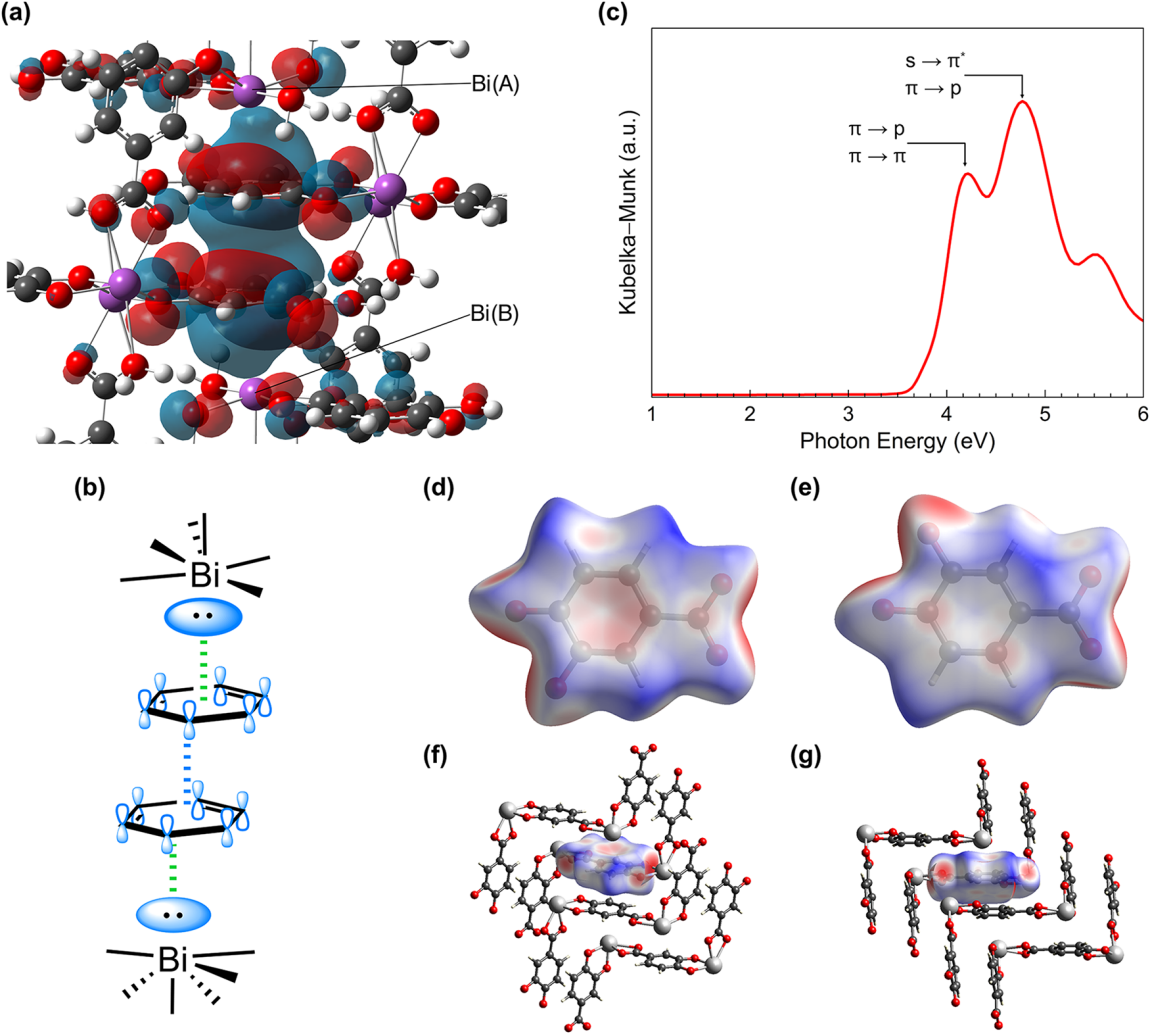
Electronic property of **1**. (a) Lone pair−π and π–π interactions depicted from the HOMO–30 molecular orbital for model 2. The Bi sites involved in the discussion of the orbital contribution analysis were designated as Bi­(A) and Bi­(B) (see main text). (b) Schematic representation of the interlayer lone pair−π and π–π interactions based on the X-ray and theoretical studies. (c) Diffuse reflectance spectrum at rt. (d–g) Hirshfeld surface of PCA^3–^ ligand for lone pair−π side (panels d, f) and π–π interaction side (panels e, g). The Hirshfeld surfaces were mapped with *d*_norm_ over the range of −0.8 to +1.0, where *d*_norm_ is a normalized contact distance.

Further evidence for these intermolecular interactions was obtained through diffuse reflectance spectroscopy at rt, as shown in Figure 5c, which revealed absorption bands that can be attributed to lone pair−π interactions. Three characteristic broad absorption bands were observed at 4.2, 4.8, and 5.5 eV. On the basis of the results of TD-DFT calculation, the absorption band observed at 4.2 eV can be assigned to π–π* transitions within the aromatic ring of the PCA^3–^ ligand together with ligand-to-metal charge-transfer (LMCT) transitions from the aromatic ligand π orbitals to the Bi^3+^ p-orbital. Meanwhile, the absorption band at 4.8 eV is attributable to LMCT transitions from the aromatic ligand π orbital to the Bi^3+^ p-like orbital, as well as metal-to-ligand charge-transfer (MLCT) transitions from the Bi^3+^ s-like orbitals to the aromatic ligand π* orbitals. Such electronic transitions involving the Bi^3+^ orbitals and the aromatic rings of the ligands are considered to represent characteristic transitions reflecting the lone pair−π interaction in **1** (details of the assignments of the absorption bands are summarized in Table S4 and Figure S9). These experimental and theoretical results confirm that the observed optical features originate from interactions between the Bi^3+^ ions and the aromatic systems of the PCA^3–^ ligands in **1**.

Moreover, to visualize the extent to which the PCA^3–^ ligands interact with the surrounding atomic species in **1**, a Hirshfeld surface analysis was performed.^46^ The results of the Hirshfeld surface analysis for **1** are shown in Figure 5d–g. In these figures, regions of strong intermolecular interactions are shown in red, whereas those of weak interactions are shown in blue. On the surface where the PCA^3–^ ligand interacts with Bi^3+^ (Figure 5d), the interacting regions appear in red; in contrast, on the surface corresponding to the π–π interaction side (Figure 5e), the interacting regions are displayed in white. As clearly demonstrated in the packing-model-extended Hirshfeld surface mappings (Figure 5f,g), in **1**, the interaction between the 6s^2^ lone pair of Bi^3+^ and the π-electron cloud of the PCA^3–^ ligand (i.e., lone pair−π interaction) is stronger than the π–π interactions between neighboring PCA^3–^ ligands. This interaction effectively contributes to the stabilization of the overall crystal structure, in which two-dimensional layered structures are stacked.

## Conclusion

In conclusion, we have successfully synthesized a novel layered Bi-based CP, **1**, which represents the first example of a MOF or CP incorporating the cation-based lone pair−π interaction. Detailed single-crystal X-ray diffraction analysis revealed that **1** possesses an interdigitated 2D layered structure, where adjacent layers are linked through both lone pair−π and π–π interactions. Furthermore, DFT calculations and spectroscopic observations confirmed the presence of relatively strong intermolecular interactions between the 2D layers and the existence of characteristic absorption bands arising from the lone pair−π interaction. MOFs and CPs characterized by intermolecular interactions arising from such unusual coordination environments may offer significantly greater structural diversity compared with conventional framework compounds composed of commonly studied transition-metal ions. Furthermore, the use of post-transition-metal ions, whose stereochemical activity can be flexibly modulated by the coordination number and ligand environment, may enable the development of functional materials in which structural flexibility is directly coupled with electronic properties, thereby opening possibilities for applications such as guest-responsive sensor materials.

## Experimental Section

### Materials

All reagents, Bi­(NO_3_)_3_·5H_2_O, 3,4-dyhydroxybenzoic acid (protocatechuic acid (H_3_PCA)), acetic acid, and solvents were purchased from Kanto Chemical Co., Inc., Japan, and Tokyo Chemical Industry (TCI) Co., Ltd., Japan, and were used without further purification.

### Physical Measurements

A Raman spectrum was recorded with a JASCO NRS-5100 spectrometer at rt. A Nd:YAG laser provided the exciting line (*E*_i_ = 532 nm). A Powder X-ray diffraction (PXRD) pattern was collected using a Rigaku MiniFlex600-C X-ray diffractometer (Cu Kα radiation) at rt. A Fourier-transform infrared (FTIR) spectrum was measured with a JASCO FT/IR-4x spectrometer at rt. Thermogravimetric analysis (TGA) on an as-synthesized polycrystalline powder sample was performed with a Rigaku Thermo plus EVO2 at a heating rate of 5 K/min in a constant flow of N_2_ gas. A Diffuse reflectance spectrum diluted in CaF_2_ powder was recorded using a JASCO V-570 spectrometer with an ISN-470 60 mm ϕ integrating sphere apparatus at rt. Elemental analysis was performed using an MT-6 CHN recorder (Yanaco) at the Organic Microanalysis Laboratory, Kyoto University.

### Synthesis of Bi­(PCA)­(H_2_O) (1)

30.0 mg (0.062 mmol) of Bi­(NO_3_)_3_·5H_2_O was dissolved in 5 mL of H_2_O, and 0.4 mL of acetic acid was added to this solution. 28.6 mg (0.186 mmol) of H_3_PCA was dissolved in 5 mL H_2_O. These solutions were mixed slowly in a screw-capped vial (20 mL). This mixture was heated in the temperature-programmable oven to 80 °C for 12 h, held at this temperature for 96 h, then cooled to rt for 12 h. Yellow hexagonal platelet crystals (Figure S1) suitable for single-crystal XRD measurements were obtained. Elemental analysis (% calcd, % found for C_7_H_5_BiO): C (22.24, 22.47), H (1.33, 1.52).

### Single-Crystal X-ray Diffraction

Single-crystal XRD measurements were carried out using a Bruker D8 QUEST PHOTON III detector with graphite-monochromated Mo Kα radiation (λ = 0.71073 Å) at 90 K. The single crystal was mounted on a MicroMesh (MiTeGen) with Paraton-N oil (Hampton Research). The structures were solved by the dual-space method (SHELXT version 2018/2)^47^ and refined by full-matrix least-squares refinement on *F* ^2^ (SHELXL version 2019/3)^48^ using the CrystalStructure 4.3.2. software.^49^

### Crystallographic Data

Data for Bi­(PCA)­(H_2_O) (C_7_H_5_BiO) at 90 K: *M*_r_ = 756.19, crystal dimensions 0.09 × 0.07 × 0.05 mm^3^, monoclinic, space group *P*2_1_/*n* (no. 14), *a* = 8.8209(5) Å, *b* = 9.0728(5) Å, *c* = 9.1511(5) Å, β = 103.769(2)°, *V* = 711.32(7) Å^3^, *Z* = 4, *d*_calc_ = 3.530 g/cm^3^, *F*(000) = 680.00, μ = 24.724 mm^–1^, A total of 6742 reflections were collected, of which 1259 reflections were unique (*R*_int_ = 0.0446). Refinement of 124 parameters led to the final *R*_1_ = 0.0218 (*I* > 2σ­(*I*)), *wR*_2_ = 0.0532 (all data), goodness of fit (GOF) = 1.111, and residual electron density max/min = 0.76/–1.25 eÅ^–3^. CCDC reference number 2534189.

### Computational Study

DFT calculations on structural optimization and vibrational frequency and TD-DFT calculations on electronic transitions were performed using the Gaussian09W package^50^ and the PBE1PBE functional. The SDD basis set and the effective core potential were used for Bi. The 6–31g­(d,p) basis set was used for other atoms. For details, see the Supporting Information.

## Supplementary Material



## Notes

Additional figures and tables, including Raman and FTIR spectra, PXRD pattern, and details of DFT calculations (PDF)
