# Supplementary material for: A Two-Dimensional Layered Bismuth Coordination Polymer Based on a Lone Pair−π Interaction
Source: Inorg Chem. 2026 Jun 12;65(25):14224–30. doi: 10.1021/acs.inorgchem.6c01816 (PMC13321301; doi:10.1021/acs.inorgchem.6c01816)
Supplement: Supplementary file 1 [file ic6c01816_si_001.pdf]

Supporting Information

A two-dimensional layered bismuth coordination polymer based on a lone pair–π interaction

Haruto Yamaoka,[†] and Kazuya Otsubo*[,†,‡]

[†]Department of Chemistry, Faculty of Science, Tokyo University of Science, Shinjuku-ku, Tokyo, 162-8601, Japan

[‡]Research Institute for Science and Technology, Division of Joint Research of Geometry and Natural Science, Tokyo University of Science, Chiba, 278-8510, Japan

*e-mail: otsubok@rs.tus.ac.jp

Contents

Figures S1–S9 and Tables S1–S4 .... pp. S2–S17

Figures S1–S9 and Tables S1–S4

Table S1. Crystal data and structure refinement of **1**.

Empirical Formula	C7 H5 Bi O5
Formula weight	378.09
Temperature	90(2) K
Wavelength	0.71073 Å
Crystal system	monoclinic
Space group	$P2_1/n$ (#14)
Unit cell dimensions	a = 8.8209(5) Å, α = 90°.
	b = 9.0728(5) Å, β = 103.769(2)°.
	c = 9.1511(5) Å, γ = 90°.
Volume (V)	711.32(7) $Å^3$
Z	4
Density (calculated)	3.530 g/cm^3
Absorption coefficient	24.724 mm^{-1}
F(000)	680
Crystal size	0.090 × 0.070 × 0.050 mm^3
Theta range for data collection	2.883 to 25.147°
Index ranges	$-10 \leq h \leq 10$, $-10 \leq k \leq 10$, $-10 \leq l \leq 10$
Reflections collected	6742
Independent reflections	1259 (R_{int} = 0.0446)
Completeness to theta = 25.147°	98.8%
Absorption correction	multi-scan
Max. and min. transmission	0.187 and 0.372
Refinement method	Full-matrix least-squares on F^2
Data/restraints/parameters	1259/3/124
Goodness-of-fit on F^2	1.111
Final R indices [I>2σ(I)]	R_1 = 0.0218, wR_2 = 0.0527
R indices (all reflections)	R_1 = 0.0228, wR_2 = 0.0532
Largest diff. peak and hole	0.76 and –1.25 $eÅ^{-3}$
CCDC reference number	2534189

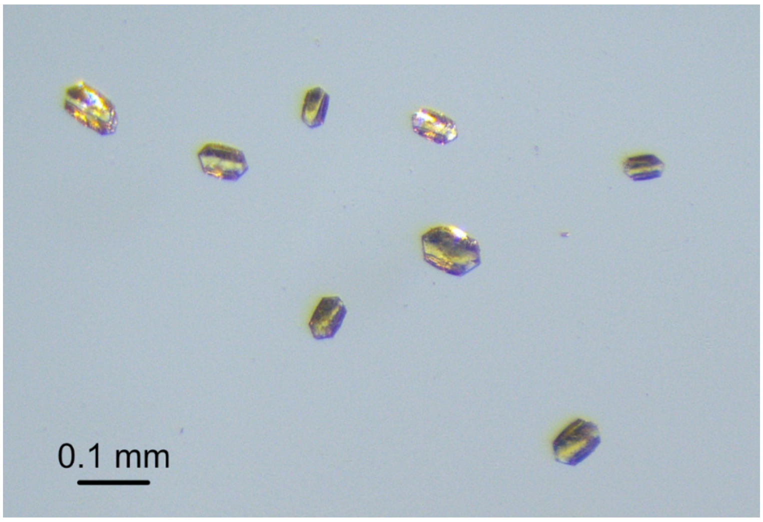

Figure S1. Single crystals of **1**.

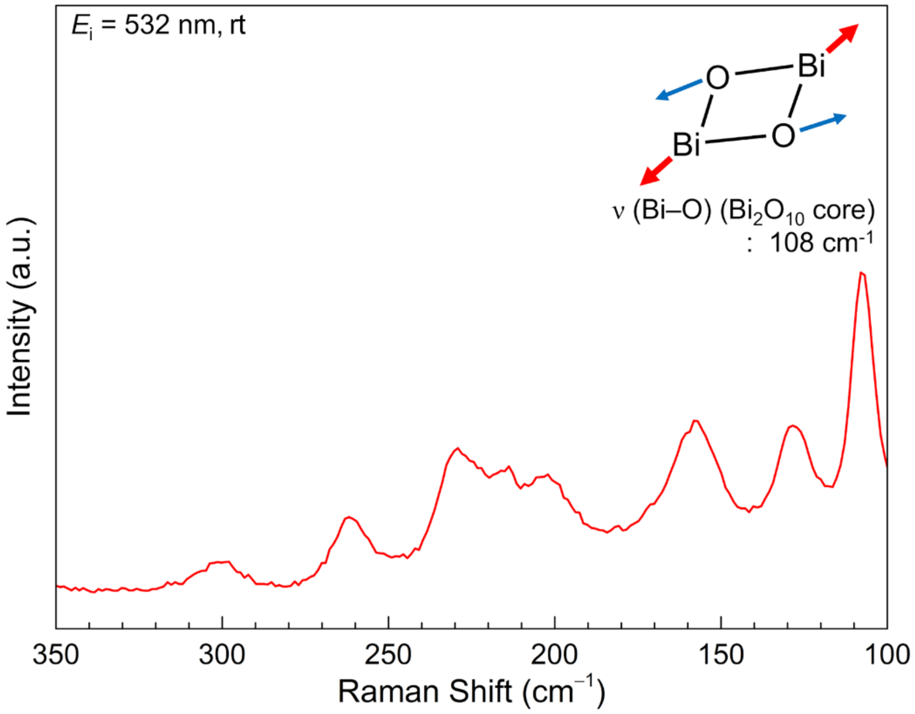

Figure S2. Raman spectrum of **1** at rt (E_i = 532 nm).

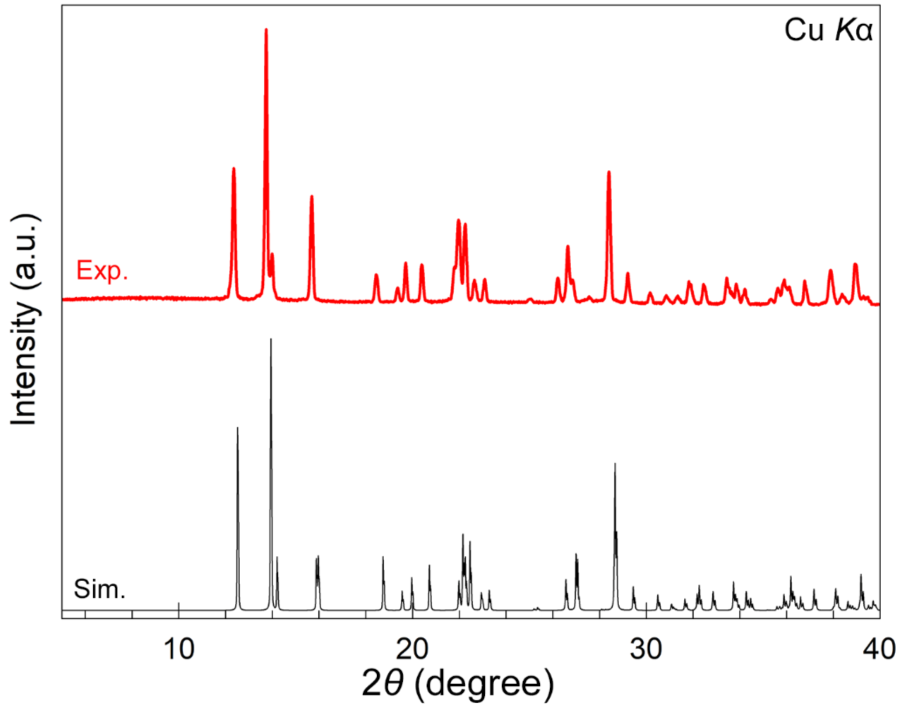

Figure S3. Powder X-ray diffraction patterns of as-synthesized **1** (red) and simulated **1** based on the single crystal X-ray analysis (black) at rt.

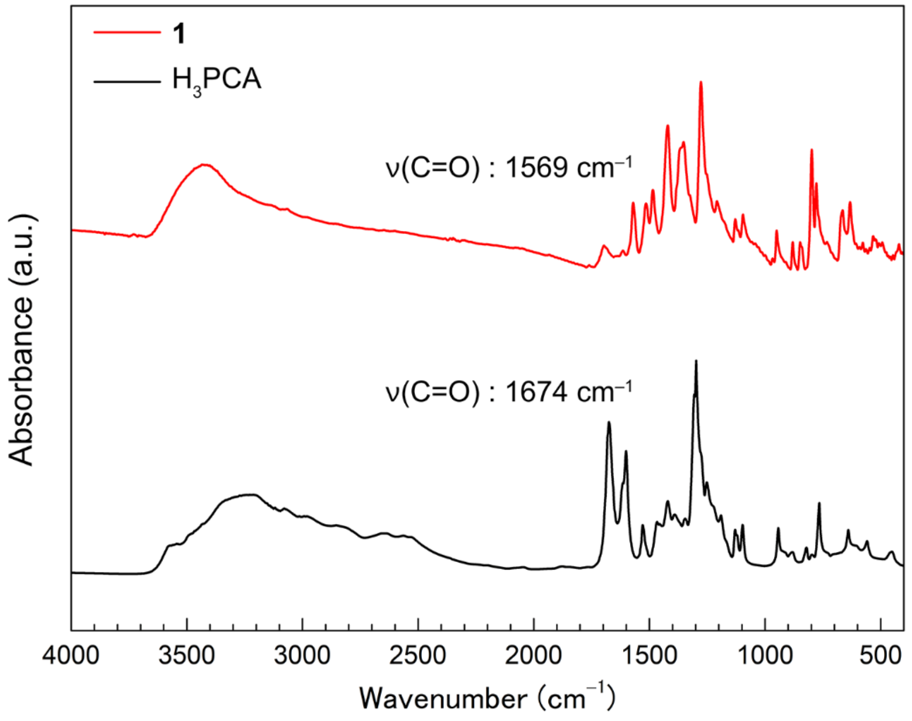

Figure S4. FTIR spectra of **1** and free H_3PCA at rt.

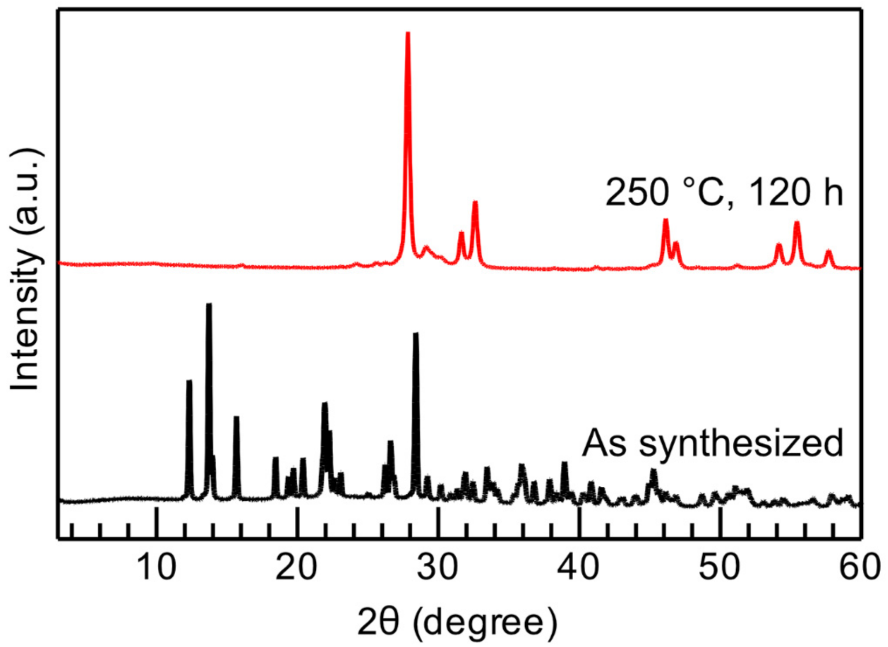

Figure S5. PXRD patterns of **1** and **1** after heated at 250 °C for 120 h.

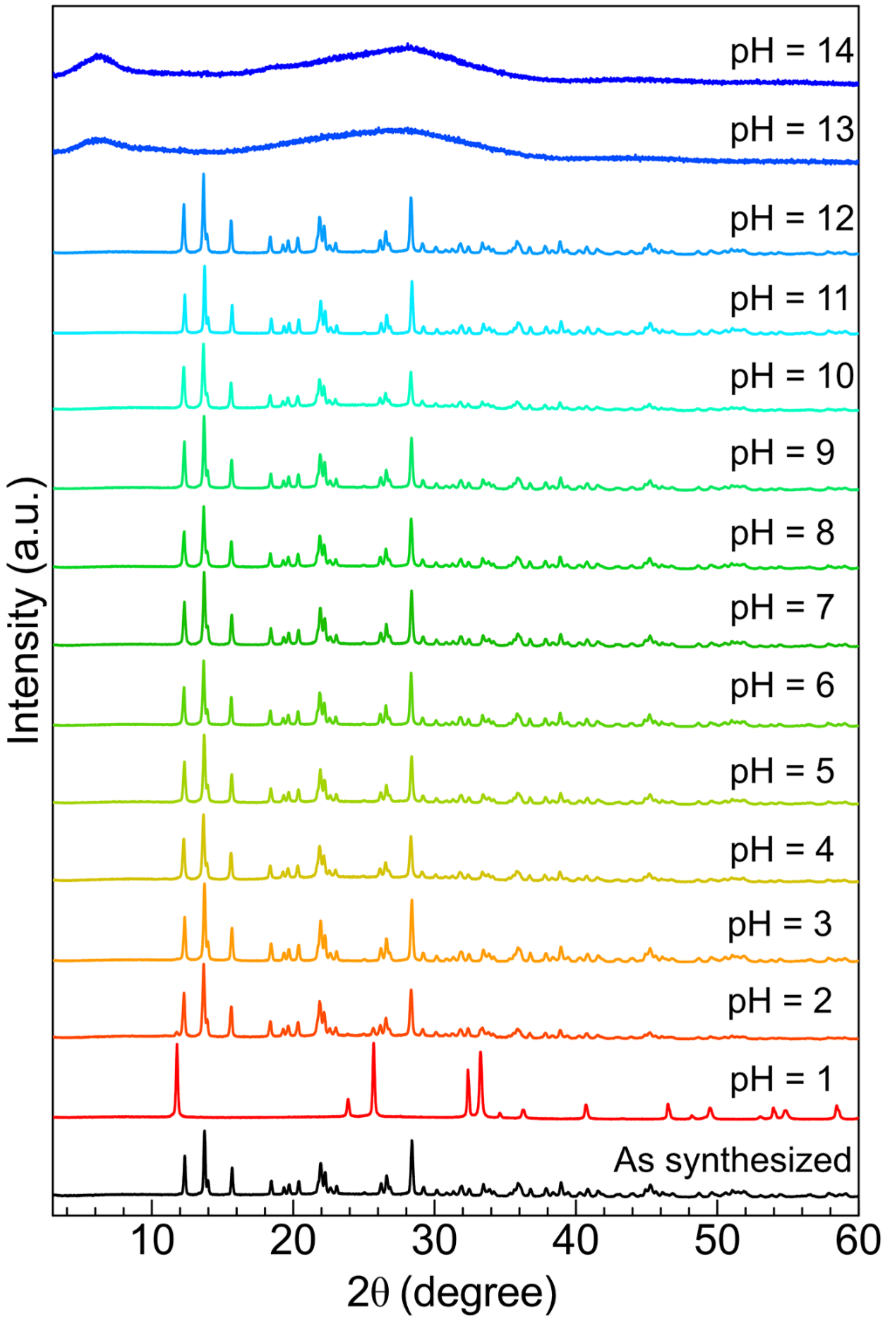

Figure S6. PXRD patterns of **1** in different stats. Polycrystalline powder samples of **1** were immersed for 24 h in a series of aqueous solutions with pH values adjusted using HCl and NaOH. The samples were subsequently collected by filtration, dried, and characterized by PXRD measurements.

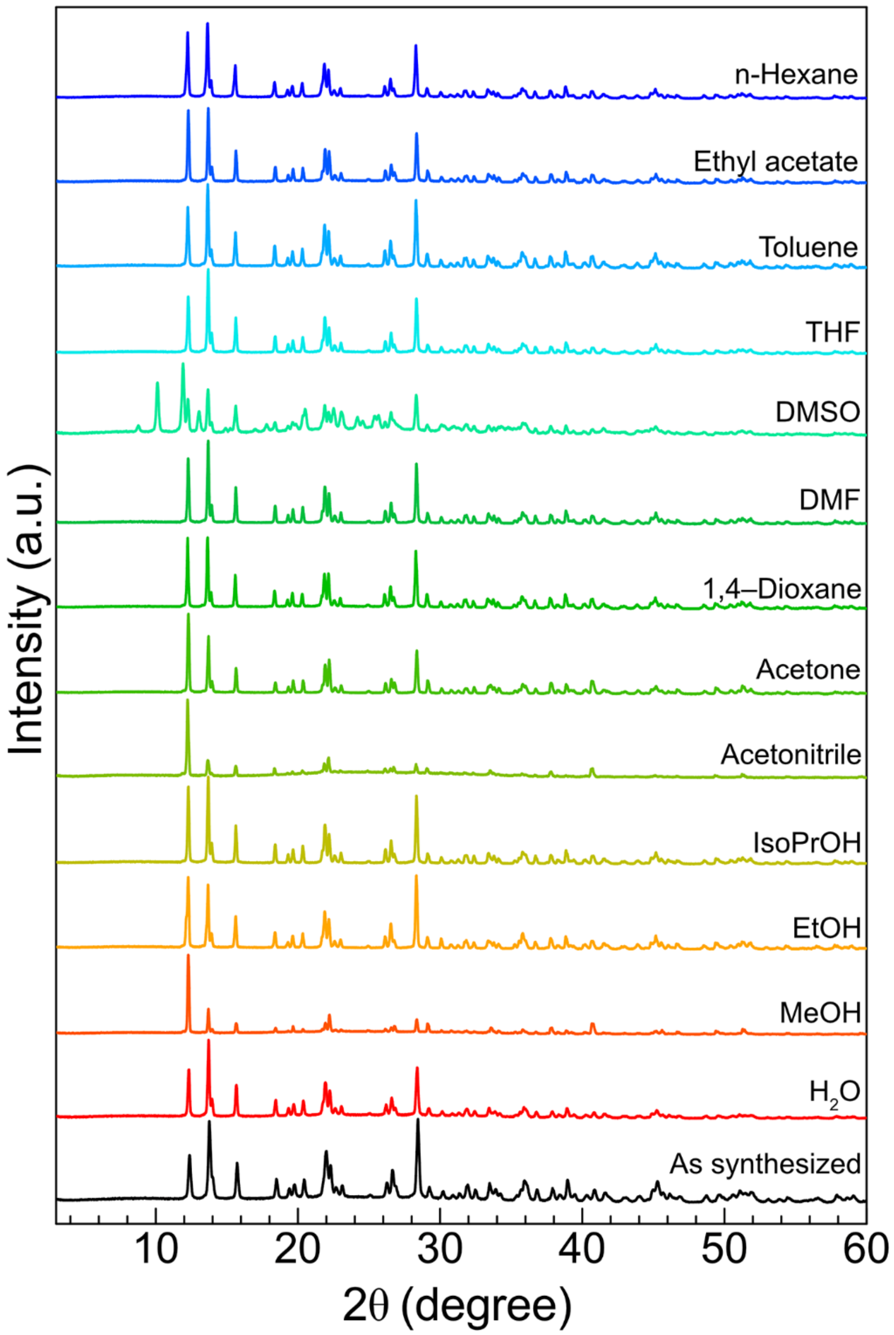

Figure S7. PXRD patterns of **1** in different stats. Polycrystalline powder samples of **1** were also immersed in various solvents for 24 h, after which the samples were isolated by filtration, dried, and analyzed by PXRD measurements.

Table S2. Cartesian coordinates for the DFT calculation.

(Model 1, single-Bi_2O_{10}-core model).

Center Number	Atomic Number	Atomic Type	Coordinates (Å) X	Y	Z
1	83	0	0.763554	0.099882	–1.761817
2	83	0	–0.763660	–0.100084	1.761823
3	8	0	0.426824	–1.885021	–2.646359
4	8	0	–0.426543	1.884731	2.646402
5	8	0	0.135868	–7.141575	0.827217
6	8	0	–0.135463	7.141347	–0.827068
7	8	0	–3.003137	0.628190	1.864957
8	8	0	3.003200	–0.627963	–1.865144
9	8	0	0.450824	–1.208170	–0.049714
10	8	0	–0.451016	1.207958	0.049724
11	8	0	0.174590	–5.623609	2.473963
12	8	0	–0.174626	5.623438	–2.473856
13	8	0	–2.564992	–0.754663	0.213466
14	8	0	2.564763	0.754470	–0.213383
15	8	0	–1.657155	–0.213006	–2.227004
16	8	0	1.657074	0.212396	2.227185
17	6	0	0.383004	–2.534289	–0.369978
18	6	0	–0.382978	2.534056	0.370030
19	6	0	0.383528	–2.865671	–1.748207
20	6	0	–0.383241	2.865394	1.748269
21	6	0	0.329595	–3.520738	0.594349
22	6	0	–0.329594	3.520527	–0.594277
23	6	0	–5.272681	–0.803138	–0.589029
24	6	0	5.272484	0.803507	0.589003
25	1	0	0.358608	–3.280946	1.653218
26	1	0	–0.358808	3.280764	–1.653147
27	1	0	–4.571325	–1.458685	–1.098388
28	1	0	4.571001	1.458838	1.098464
29	6	0	0.321590	–4.214301	–2.114034
30	6	0	–0.321086	4.214007	2.114122
31	1	0	0.330373	–4.460047	–3.171079
32	1	0	–0.329681	4.459722	3.171176
33	6	0	0.187371	–5.872611	1.284788
34	6	0	–0.187195	5.872403	–1.284672
35	6	0	–3.406137	–0.037018	0.864019
36	6	0	3.406076	0.037165	–0.864109
37	6	0	0.253254	–5.205498	–1.143795
38	6	0	–0.252785	5.205226	1.143903
39	1	0	0.203681	–6.249522	–1.433357
40	1	0	–0.203045	6.249235	1.433488
41	6	0	0.254441	–4.867127	0.211969
42	6	0	–0.254213	4.866896	–0.211872
43	6	0	–4.819024	0.004471	0.464372
44	6	0	4.818973	–0.004088	–0.464472
45	1	0	–1.560694	–1.083337	–2.640778
46	1	0	1.560611	1.082760	2.640896
47	1	0	–2.123041	–0.367626	–1.369781

48	1	0	2.122919	0.366992	1.369941
49	8	0	7.166010	1.498034	1.958748
50	8	0	–7.166329	–1.497432	–1.958723
51	6	0	–6.601814	–0.761462	–0.957523
52	6	0	6.601639	0.762085	0.957451
53	6	0	–5.711025	0.842535	1.136679
54	6	0	5.711127	–0.841926	–1.136856
55	6	0	–7.501475	0.085900	–0.282353
56	6	0	7.501457	–0.085039	0.282194
57	6	0	–7.047220	0.884254	0.763145
58	1	0	–5.339308	1.458051	1.948421
59	1	0	5.339523	–1.457438	–1.948652
60	6	0	7.047336	–0.883415	–0.763345
61	8	0	–8.794658	0.118956	–0.649065
62	8	0	8.794661	–0.117844	0.648859
63	1	0	–7.758560	1.529457	1.267548
64	1	0	7.758798	–1.528422	–1.267827
65	1	0	0.096614	–7.694732	1.619911
66	1	0	–0.096263	7.694522	–1.619753
67	1	0	6.500747	2.050886	2.378834
68	1	0	8.906603	0.499503	1.384644
69	1	0	–8.906662	–0.498268	–1.384944
70	1	0	–6.500907	–2.049471	–2.379625

Table S3. Cartesian coordinates for the TDDFT calculation.

(Model 2, quadruple-Bi_2O_{10}-core model)

Center Number	Atomic Number	Atomic Type	Coordinates (Å) X	Y	Z
1	6	0	4.173792	6.114779	2.506467
2	6	0	2.816679	5.893146	2.716275
3	1	0	4.552522	6.917643	2.784083
4	1	0	2.293422	6.544069	3.124006
5	6	0	–7.522847	1.287374	2.625491
6	6	0	–8.879961	1.065742	2.835299
7	1	0	–7.144117	2.090239	2.903107
8	1	0	–9.402664	1.717187	3.242381
9	83	0	–1.945954	4.016093	2.948097
10	6	0	–2.694532	6.376060	1.256289
11	6	0	–2.900314	6.855129	2.564268
12	6	0	–2.910731	7.190867	0.166351
13	6	0	–7.793279	3.860881	0.311144
14	6	0	–3.317045	8.166598	2.733125
15	6	0	–3.676176	9.332381	–0.852447
16	6	0	0.786629	4.422252	2.469547
17	6	0	–3.563345	8.993518	1.641134
18	6	0	–3.373593	8.495710	0.329050
19	6	0	–6.975439	2.890414	–0.266025
20	1	0	–4.920505	3.258699	3.624875
21	1	0	–4.244320	2.184617	3.594189
22	1	0	–2.745000	6.866206	–0.689132
23	1	0	–7.431000	4.683957	0.547834
24	1	0	–3.435679	8.501158	3.593600
25	1	0	–3.849513	9.867535	1.772553
26	8	0	–2.715129	6.010090	3.600307
27	8	0	–4.003461	10.563106	–0.724721
28	8	0	0.039261	5.256440	3.089583
29	8	0	–2.322060	5.060542	1.115782
30	8	0	–3.585553	8.842405	–2.023885
31	8	0	–5.003446	4.205101	0.024268
32	8	0	–4.346917	3.003474	2.901999
33	6	0	–10.910010	–0.405152	2.588571
34	8	0	–11.017263	2.182315	0.427790
35	8	0	–11.656825	0.429558	3.207959
36	6	0	–9.131361	3.616687	0.535977
37	6	0	–9.713165	2.394176	0.150999
38	8	0	–9.934782	4.524706	1.183069
39	6	0	8.720935	–1.093194	4.980791
40	6	0	4.390059	3.940764	1.505725
41	6	0	8.515500	–0.614978	6.288379
42	6	0	4.972417	5.163798	1.890055
43	6	0	8.504735	–0.278388	3.890852
44	6	0	8.098422	0.697344	6.457627
45	6	0	8.914249	–1.441716	0.464976
46	6	0	7.739291	1.863127	2.872055
47	6	0	7.852121	1.524263	5.365635

48	6	0	7.557483	–1.664201	0.674394
49	6	0	8.041873	1.026456	4.053552
50	1	0	8.670466	–0.603049	3.035370
51	1	0	7.980136	1.031051	7.317712
52	1	0	9.292978	–0.638852	0.742592
53	1	0	7.565953	2.398281	5.497055
54	1	0	7.033879	–1.012426	1.082515
55	8	0	8.700337	–1.459165	7.324809
56	8	0	7.412006	3.093852	2.999781
57	8	0	9.093406	–2.408712	4.840284
58	8	0	5.194033	3.033268	0.857984
59	8	0	7.830261	1.372298	1.700227
60	8	0	4.815051	0.348631	1.586696
61	6	0	9.130515	–3.615731	–0.535766
62	6	0	9.713221	–2.393550	–0.151826
63	8	0	9.934489	–4.523227	–1.183507
64	83	0	7.258775	3.582805	0.711331
65	6	0	8.643817	1.131791	–1.849371
66	6	0	9.461656	0.161323	–2.426540
67	1	0	7.031645	5.416951	–1.830128
68	1	0	7.892340	4.518219	–2.077066
69	1	0	9.006096	1.954866	–1.612681
70	8	0	6.275961	5.375137	1.613912
71	8	0	11.433096	1.475488	–2.135598
72	6	0	10.909717	0.406631	–2.589009
73	8	0	11.016417	–2.181358	–0.427579
74	8	0	11.656532	–0.428079	–3.208396
75	6	0	–2.975704	–5.920598	5.099815
76	6	0	–7.306027	–0.886118	1.624101
77	6	0	–3.180586	–5.441860	6.406755
78	6	0	–6.724223	0.336393	2.009079
79	6	0	–3.191904	–5.105792	4.009876
80	6	0	–3.597461	–4.130050	6.577305
81	6	0	–3.957349	–2.964278	2.991079
82	6	0	–3.843761	–3.303131	5.485313
83	6	0	–3.654213	–3.800426	4.171927
84	1	0	–3.025620	–5.429930	3.153745
85	1	0	–3.716503	–3.796353	7.436736
86	1	0	–4.130686	–2.429124	5.616079
87	8	0	–2.996302	–6.286569	7.443833
88	8	0	–4.284633	–1.733552	3.118805
89	8	0	–2.603233	–7.236116	4.959308
90	8	0	–6.502606	–1.794137	0.977008
91	8	0	–3.866378	–3.455106	1.819251
92	8	0	–6.881589	–4.478774	1.705720
93	83	0	–7.259067	–3.581326	–0.711768
94	6	0	–8.644109	–1.130312	1.848934
95	6	0	–9.461192	–0.159834	2.426757
96	1	0	–7.031589	–5.416324	1.829301
97	1	0	–7.891876	–4.516730	2.077282
98	1	0	–9.006389	–1.953387	1.612244
99	8	0	–6.276806	–5.374180	–1.613701
100	8	0	–11.433942	–1.474532	2.135810
101	83	0	2.794502	–3.540402	0.906606
102	83	0	4.437572	1.246078	–0.830792

103	83	0	−2.795348	3.541358	−0.906395
104	83	0	−4.437108	−1.244589	0.831008
105	6	0	2.045925	−1.180435	−0.785201
106	6	0	−2.046217	1.181914	0.784764
107	6	0	1.840490	−0.702219	0.522387
108	6	0	−1.840988	0.702323	−0.522565
109	6	0	1.829725	−0.365628	−1.875140
110	6	0	−3.052823	−3.695614	−1.730347
111	6	0	−1.830018	0.367107	1.874703
112	6	0	3.052530	3.697093	1.729910
113	6	0	1.424168	0.610113	0.692288
114	6	0	−1.423704	−0.608624	−0.692072
115	6	0	1.064281	1.775886	−2.893938
116	6	0	−5.527378	3.135722	−0.428494
117	6	0	−1.064573	−1.774407	2.893501
118	6	0	5.527086	−3.134243	0.428056
119	6	0	1.177868	1.437033	−0.399703
120	6	0	−1.177404	−1.435544	0.399920
121	6	0	1.366863	0.939215	−1.712441
122	6	0	−2.234983	−4.666081	−2.307516
123	6	0	−1.367709	−0.938259	1.712652
124	6	0	2.234690	4.667560	2.307079
125	1	0	−0.179700	−4.298649	1.582994
126	1	0	4.664496	−0.589442	1.710925
127	1	0	0.179756	4.299275	−1.583821
128	1	0	−4.664994	0.589546	−1.711104
129	1	0	0.496136	−5.371878	1.552698
130	1	0	3.804007	0.310665	1.957605
131	1	0	−0.496429	5.373357	−1.553136
132	1	0	−3.804299	−0.309186	−1.958042
133	1	0	1.995456	−0.690289	−2.730623
134	1	0	−2.690543	−2.872538	−1.493657
135	1	0	−1.996302	0.691246	2.730834
136	1	0	2.690251	2.874017	1.493220
137	1	0	1.305126	0.943810	1.551719
138	1	0	−1.305070	−0.943184	−1.552547
139	1	0	0.890943	2.311040	−0.268938
140	1	0	−0.891236	−2.309561	0.268500
141	8	0	2.025327	−1.546405	1.558816
142	8	0	5.419833	−0.546776	−1.732724
143	8	0	−2.025620	1.547884	−1.559254
144	8	0	−5.420125	0.548255	1.732287
145	8	0	0.736996	3.006611	−2.766212
146	8	0	−4.780564	2.301011	−1.047881
147	8	0	−0.737288	−3.005132	2.765775
148	8	0	4.779718	−2.300055	1.048092
149	8	0	2.418396	−2.495953	−0.925709
150	8	0	−2.417932	2.497442	0.925925
151	8	0	1.155251	1.285058	−4.065766
152	8	0	−0.262990	−3.351394	−2.017223
153	8	0	−1.155196	−1.284431	4.064939
154	8	0	0.262698	3.352873	2.016786
155	8	0	0.393539	−4.553021	0.860509
156	8	0	−0.393831	4.554500	−0.860946
157	83	0	1.945108	−4.015137	−2.947886

158	6	0	2.694239	–6.374581	–1.256727
159	6	0	2.899468	–6.854172	–2.564056
160	6	0	2.910439	–7.189388	–0.166788
161	6	0	7.792986	–3.859402	–0.311581
162	6	0	3.316752	–8.165119	–2.733563
163	6	0	–0.786165	–4.420763	–2.469331
164	6	0	3.675883	–9.330902	0.852010
165	6	0	3.563053	–8.992039	–1.641571
166	6	0	3.372747	–8.494754	–0.328839
167	6	0	6.975147	–2.888935	0.265588
168	1	0	4.920212	–3.257219	–3.625312
169	1	0	4.244028	–2.183138	–3.594627
170	1	0	2.744154	–6.865249	0.689343
171	1	0	7.430707	–4.682478	–0.548271
172	1	0	3.435386	–8.499679	–3.594038
173	1	0	3.849569	–9.866908	–1.773380
174	8	0	2.714837	–6.008611	–3.600745
175	8	0	–0.040107	–5.255484	–3.089372
176	8	0	4.003516	–10.562480	0.723894
177	8	0	2.322525	–5.059053	–1.115566
178	8	0	3.585261	–8.840926	2.023448
179	8	0	5.003502	–4.204475	–0.025095
180	8	0	4.346625	–3.001995	–2.902437
181	6	0	7.305734	0.887597	–1.624538
182	6	0	2.975412	5.922077	–5.100252
183	6	0	6.723930	–0.334914	–2.009516
184	6	0	3.180641	5.442486	–6.407582
185	6	0	3.191058	5.106749	–4.009665
186	6	0	3.597372	4.131017	–6.576440
187	6	0	3.957056	2.965757	–2.991516
188	6	0	3.844225	3.304620	–5.485097
189	6	0	3.653920	3.801905	–4.172364
190	1	0	3.025327	5.431410	–3.154182
191	1	0	3.716559	3.796980	–7.437563
192	1	0	4.130393	2.430603	–5.616516
193	8	0	2.996010	6.288048	–7.444270
194	8	0	4.284341	1.735032	–3.119242
195	8	0	6.502314	1.795616	–0.977446
196	8	0	2.603697	7.237605	–4.959091
197	8	0	3.866433	3.455732	–1.820078
198	8	0	6.881296	4.480253	–1.706157
199	6	0	7.522554	–1.285895	–2.625928
200	6	0	8.879668	–1.064263	–2.835736
201	1	0	7.143619	–2.090135	–2.903285
202	1	0	9.402719	–1.716560	–3.243208
203	6	0	–4.390905	–3.939808	–1.505514
204	6	0	–8.721227	1.094673	–4.981228
205	6	0	–4.972709	–5.162319	–1.890492
206	6	0	–8.515998	0.615082	–6.288558
207	6	0	–8.505028	0.279867	–3.891290
208	6	0	–8.914541	1.443195	–0.465414
209	6	0	–8.098714	–0.695865	–6.458064
210	6	0	–7.739583	–1.861648	–2.872492
211	6	0	–7.557427	1.664828	–0.675222
212	6	0	–7.852414	–1.522784	–5.366073

213	6	0	–8.042719	–1.025500	–4.053341
214	1	0	–8.671312	0.604005	–3.035158
215	1	0	–9.293271	0.640331	–0.743029
216	1	0	–7.980080	–1.030425	–7.318539
217	1	0	–7.034725	1.013383	–1.082303
218	1	0	–7.566246	–2.396801	–5.497492
219	8	0	–8.700630	1.460644	–7.325246
220	8	0	–7.412298	–3.092373	–3.000218
221	8	0	–5.194325	–3.031789	–0.858422
222	8	0	–9.092942	2.410201	–4.840067
223	8	0	–7.830206	–1.371672	–1.701054
224	8	0	–4.814586	–0.347142	–1.586479
225	6	0	–4.174085	–6.113300	–2.506904
226	6	0	–2.816971	–5.891667	–2.716712
227	1	0	–4.552467	–6.917017	–2.784910
228	1	0	–2.293920	–6.543965	–3.124184
229	1	0	8.668014	–1.524486	8.282039
230	1	0	9.131419	–2.584822	5.783226
231	1	0	4.215914	–11.176515	1.430610
232	1	0	–4.230908	11.252178	–1.353249
233	1	0	2.550630	7.467691	–5.889599
234	1	0	3.235896	5.546358	–8.004582
235	1	0	0.608172	3.267379	–3.681091
236	1	0	–0.512774	–3.677552	3.413111
237	1	0	–3.219984	–5.741290	8.201617
238	1	0	–2.480997	–7.706061	5.787444
239	1	0	–9.186123	2.778825	–5.721562
240	1	0	–8.519532	0.972495	–8.131791
241	1	0	12.560640	–0.105370	–3.215361
242	1	0	9.426028	–5.310436	–1.391759
243	1	0	11.384899	–2.959417	–0.852368
244	1	0	–12.561020	0.107086	3.214498
245	1	0	–11.385274	2.960649	0.852483
246	1	0	–9.416711	5.295978	1.424614

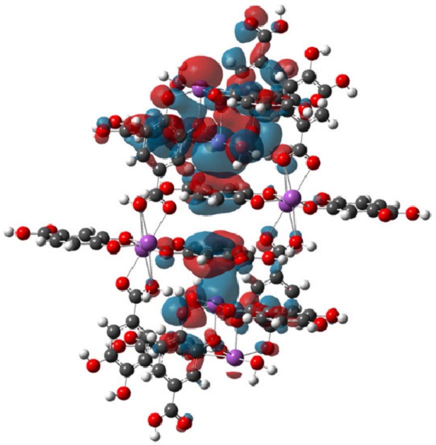
HOMO–29 (–6.973 eV)

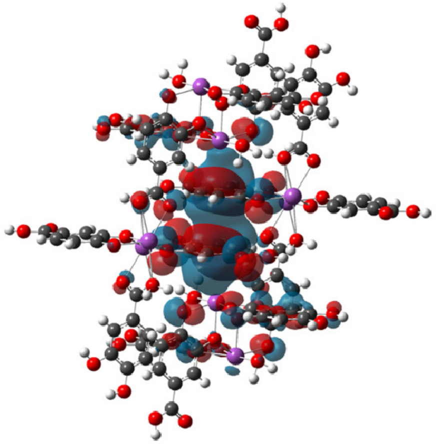
HOMO–30 (–7.145 eV)

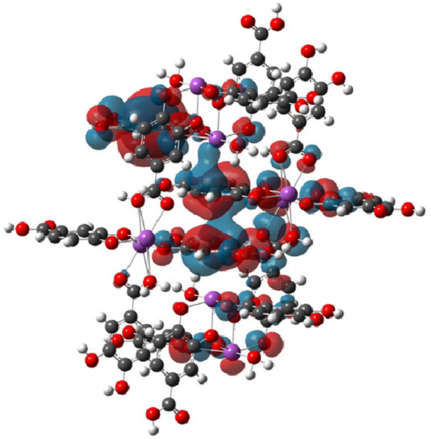
HOMO–36 (–7.336 eV)

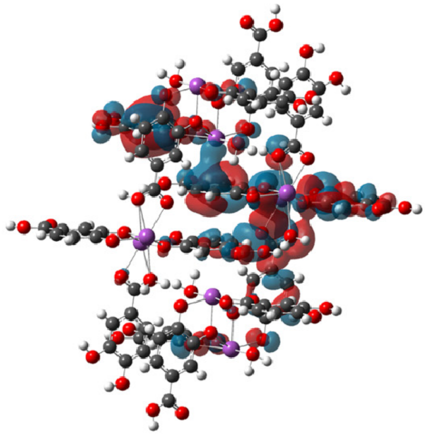
HOMO–37 (–7.362 eV)

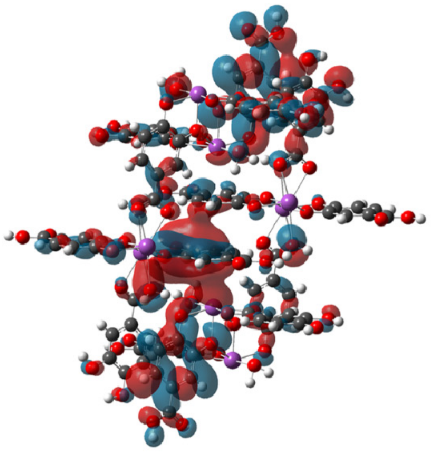
HOMO–120 (–10.304 eV)

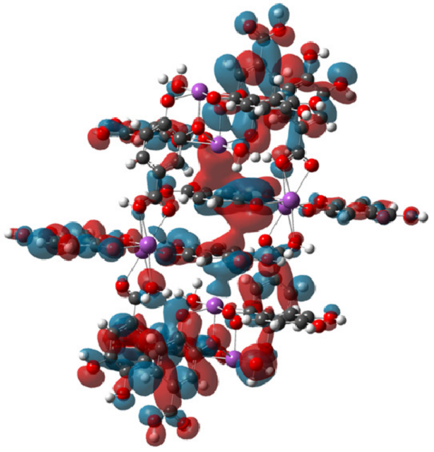
HOMO–125 (–10.385 eV)

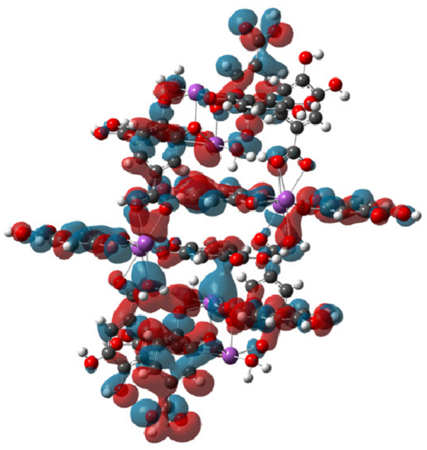
HOMO–137 (–10.726 eV)

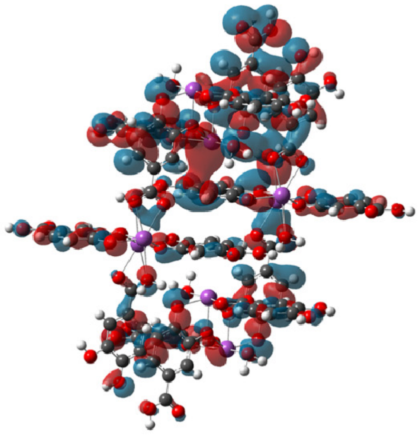
HOMO–140 (–10.789 eV)

Figure S8. Selected molecular orbitals based on the model 2 (isovalue, 0.272 eV).

Table S4. Excitation energies (*ΔE*), oscillator strengths (*f*), and assignments of excitation with relatively high probability based on the TD-DFT calculation based on model 2.

***ΔE* (eV)**	***f***	**Assignment**
3.165	0.0197	HOMO–4 → LUMO+1 (29%) (π → p(Bi))
3.273	0.0312	HOMO–3 → LUMO (38%) (π → p(Bi))
3.599	0.0116	HOMO–3 → LUMO+3 (21%) (π → π*)
4.455	0.0632	HOMO–10 → LUMO+5 (1.5%) (π → p(Bi))
4.810	0.0327	HOMO–29 → LUMO (1.9%) (s(Bi) →π*)

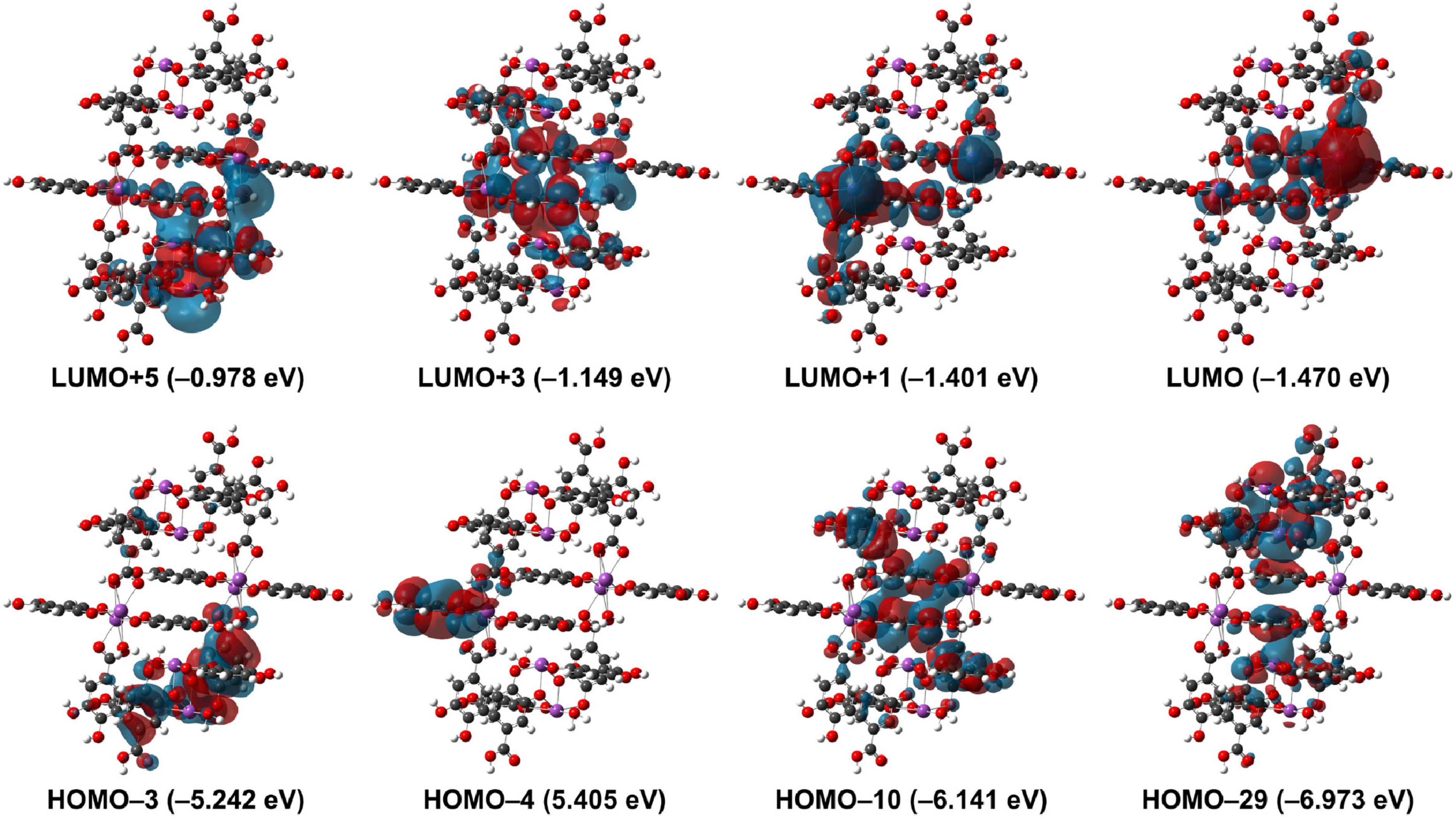

Figure S9. Selected molecular orbitals based on the model 2 regarding Table S4 (isovalue, 0.272 eV).
